# Impacts of Consumption of Ultra-Processed Foods on the Maternal-Child Health: A Systematic Review

**DOI:** 10.3389/fnut.2022.821657

**Published:** 2022-05-13

**Authors:** Priscila Gomes de Oliveira, Juliana Morais de Sousa, Débora Gabriela Fernandes Assunção, Elias Kelvin Severiano de Araujo, Danielle Soares Bezerra, Juliana Fernandes dos Santos Dametto, Karla Danielly da Silva Ribeiro

**Affiliations:** ^1^Post Graduate Program in Nutrition, Federal University of Rio Grande do Norte, Natal, Brazil; ^2^Nutrition Undergraduate Program, Federal University of Rio Grande do Norte, Natal, Brazil; ^3^Health Sciences College of Trairi, Federal University of Rio Grande do Norte, Santa Cruz, Brazil; ^4^Department of Nutrition, Federal University of Rio Grande do Norte, Natal, Brazil

**Keywords:** diet quality, pregnancy, lactation, infant, child, NOVA classification, ultra-processed food consumption, overweight/obese

## Abstract

**Background and Aims:**

Changes in eating patterns have been leading to an increase in the consumption of ultra-processed foods (UPF), negatively impacting the quality of the diet and generating risk of harm to the health of the adult population, however, there is no systematized evidence of the impact of UPF in maternal-child health. Thus, in this study we aimed to evaluated the association between UPF consumption and health outcomes in the maternal-child population.

**Methods:**

Systematic review registered on the International Prospective Register of Systematic Reviews (PROSPERO) (CRD42021236633), conducted according to the PRISMA diagram in the following databases: PubMed, Medline, Scopus, Web of Science, Scielo, and CAPES thesis and dissertation directory. We included original cross-sectional, case-control and cohort studies in any language. Eligibility criteria were (a) food consumption assessment by the NOVA classification, (b) health outcome (nutritional or diseases), and (c) maternal-child population (pregnant, lactating women and infants/children). All data were analyzed and extracted to a spreadsheet structured by two independent reviewers. We evaluated the methodological quality of the studies included using the Newcastle-Otawa Scale and RoB 2.

**Results:**

Searches retrieved 7,801 studies and 15 contemplated the eligibility criteria. Most studies included were cohort studies (*n* = 8, 53%), had children as their population (*n* = 9, 60%) and only one study evaluated UPF consumption in infants and lactating women. Panoramically, we observed that a higher participation of UPF in children’s diet has been associated with different maternal-child outcomes, such as increase of weight gain, adiposity measures, overweight, early weaning, lower diet quality, metabolic alterations, diseases, and consumption of plastic originated from packaging. Only one of the studies included did not present high methodological quality.

**Conclusion:**

Despite the limited literature on UPF consumption and health outcomes in the maternal-child population, the highest UPF consumption negatively impacted nutrition and disease development indicators in pregnant, lactating women and children. Considering the expressive participation of these foods in the diet, other studies should be conducted to further investigate the impact of UPF consumption on different health indicators, especially in the lactation phase for this was the one to present the most important knowledge gap.

**Systematic Review Registration:**

[https://www.crd.york.ac.uk/prospero/display_record.php?ID=CRD42021236633], identifier [CRD42021236633].

## Introduction

As defined by the NOVA classification, ultra-processed foods (UPF) are industrial formulations of substances derived from foods with little or no whole food and often containing added colorings, flavorings, emulsifiers, thickeners and other cosmetic additives to make them palatable or even hyperpalatable (1, 2). Despite their low nutritional quality (3), these food products are currently present in the dietary pattern of several high-income countries, representing more than half of the dietary energy consumed in countries such as the United States, Canada, and the United Kingdom (4, 5), and have also become increasingly widespread in lower-middle and upper-middle income countries (6).

In Brazil, the 2017–2018 Household Budget Survey (7) showed that this category of food represents 19.7% of the daily caloric intake of Brazilians (7), and the guidance to avoid these foods is part of the Dietary Guidelines for the Brazilian Population (8).

There is some accumulated evidence on the impact of UPF consumption on the health of the adult and elderly population, including its association with the development of Non-communicable diseases (NCDs) such as obesity, type 2 diabetes, cardiovascular disease, cancer, depression, gastrointestinal disorders, mortality from all causes, and risk of cardiometabolic diseases (9–11), but such analyses do not yet contemplate the investigation of the impact of UPF consumption in the life stages of greater biological vulnerability. Such impacts may be justified by the UPF high contents of sodium, energy, saturated and trans fats, cosmetic additives and low fiber content – important determining factors for the development of NCDs (12).

In this context, pregnancy, lactation and childhood are critical periods for growth, biological development and the establishment of eating behaviors, in addition to being a window of opportunity for health promotion and disease prevention.

During pregnancy, a greater share of UPF in the diet was associated with excessive weight gain during pregnancy (13) and an increased risk of developing gestational diabetes (14). In lactating women, a study showed the relationship between UPF consumption and breast milk composition, when a greater participation of UPF was associated with lower levels of circulating maternal alpha-tocopherol and, hypothetically, lower vitamin E supply to infants via breast milk (15). This consumption was also directly associated with the child’s eating practices (16). In childhood, studies point to the inverse relationship between UPF introduction and duration of continued breastfeeding and healthy food introduction (17).

In view of the presence of UPF in the diet of the maternal-child population (18) and known evidence about its negative impact on the health of other groups, it is necessary to systematize studies with information on UPF consumption and health outcomes in the maternal-child population. Understanding the effects of consuming these foods, provided by an overview of scientific evidence, may help to develop evidence-based policies and actions, as well as dietary recommendations to promote maternal and child health. Thus, this systematic review aimed to identify the presence of health outcomes associated with UPF consumption by pregnant women, lactating women, newborns and infants.

## Materials and Methods

The systematic review was conducted according to the diagram of Preferred Reporting Items for Systematic Reviews and Meta-Analyses (PRISMA) (19), registered in the International Prospective Register of Systematic Reviews (PROSPERO) (Identifier: CRD42021236633). The review included the following five main phases: (1) identification of the research question, (2) identification of relevant studies, (3) selection of studies, (4) data mapping, and (5) comparison, summary and reporting of results. All stages of the study were carried out independently by two reviewers (PO and JS) using a standardized collection form. When the opinion of the reviewers was conflicting, the resolution was provided by a third reviewer (KD), who also evaluated the data not included.

### Identifying the Research Question

The main objective of this review was to provide an overview of current UPF consumption practices in the maternal and child group in order to identify whether the consumption of these foods has an impact on the health outcomes of this population, based on the research question build through the PEO (Population/Problem, Exposure, and Outcome): “What are the impacts of UPF consumption on the health of the maternal and child group?” as described below.

Population (P): Pregnant, Lactating/Breastfeeding, Children (neonates, infants, and children under 10 years old).
Exposure (E): Consumption of ultra-processed foods.
Outcome (O): Diseases, nutritional status (overweight, adiposity, and weight gain), feeding practices (diet quality, unhealthy eating practices), toxicity.

### Eligibility Criteria

**FIGURE 1:**
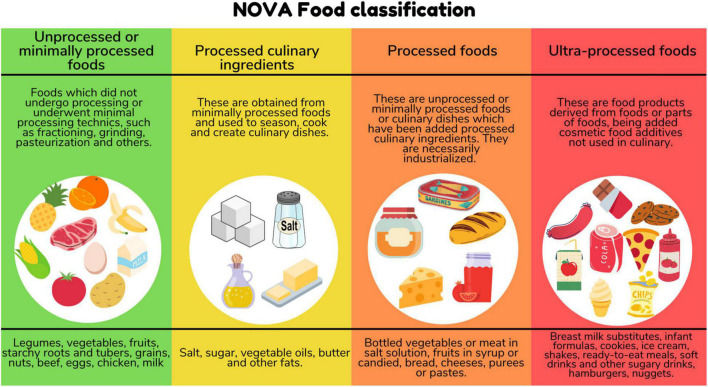
NOVA food classification.

We identified peer-reviewed publications that included the following criteria: (a) Pregnant and/or Lactating/Breastfeeding women and/or Children (neonates, infants, and children under 10 years old); (b) Percentage of total energy consumed from UPF as defined by NOVA classification (1, 2) (Figure 1), and (c) original research studies which reported health outcomes associated with UPF consumption in population describe. When we retrieved publications based on the same dataset, we included the most complete one.

### Health Outcome

We established variables related to diseases (morbidities, clinical complications), nutrition (anthropometric nutritional status, eating practices, and diet quality) and toxicity as the primary health outcomes.

### Exclusion Criteria

We excluded studies which: (1) were reviews, conference abstract, opinions, experimental studies, research protocols, clinical trials, case reports, comments, letters to editors, (2) did not assess dietary patterns using the NOVA classification, (3) did not include the separate analysis of the population of interest (pregnant women, lactating women, infants, or children), (4) did not present health outcomes that would allow us to observe the association with UPF consumption.

### Search Strategy

The search for articles happened on July 9, 2021 in the following databases: PubMed/Medline, Web of Science, Scopus, Scielo, and CAPES thesis and dissertation directory (exclusively for Brazilian studies). A new search in the same databases was carried out on September 28, 2021 to include possible new publications. MeSH descriptors that included synonyms of UPF consumption, health outcomes, pregnant women, lactating women, infants, and children in different combinations of health descriptors were used, as shown in Table 1. We applied no language limitations to the records searched. Since the NOVA classification was first proposed in 2010, no studies were available prior to that year. Brazilian thesis and dissertation research were identified by searching abstracts in the CAPES thesis and dissertation directory. Missing data were sought via emails to the corresponding authors or from secondary publications of the same study. Records were downloaded to Excel, added to a standardized data collection form and duplicates were removed.

**TABLE 1:** Search equations for systematic review conduction.

Search equation with MeSH descriptors
**Population:** Pregnancy [MESH terms] Pregnancies OR Gestation OR Pregnant Women [MESH terms] Pregnant Woman OR Woman, Pregnant Women OR Pregnant OR Lactation [MESH terms] Milk Secretion OR Milk Secretions OR Lactation, Prolonged OR Lactations Prolonged OR Prolonged Lactation OR Prolonged Lactations Breastfed OR Breast Feeding [MESH terms] Breastfeeding OR Breast Fed OR Milk Sharing OR Sharing, Milk Breast Feeding, Exclusive OR Exclusive Breast Feeding OR Breastfeeding, Exclusive OR Exclusive Breastfeeding OR Wet Nursing OR INFANT” [MESH] (Infants) Newborn [MESH terms] OR Newborn Infants OR Newborn OR Newborn Infant OR Newborn Infants OR Newborns OR Neonate OR Neonates OR CHILD [MESH terms] Children OR child, preschool.
**AND**
**Exposure:** “Ultra-processed food” “NOVA” “Food-Processing Industry” [MESH terms] (Food Processing Industry OR Industry, Food-Processing OR Food Processing Industries OR Industries, Food-Processing OR Industry, Food Processing).
**AND**
**Outcome:** Outcome Assessment, Health Care [MESH terms] Outcomes Assessment OR Outcome Assessment (Health Care) OR Assessment, Outcome (Health Care) OR Assessments, Outcome (Health Care) OR Outcome Assessments (Health Care) OR Assessment, Outcomes OR Assessments, Outcomes OR Outcomes Assessments OR Outcomes Research OR Research, Outcomes OR Outcome Studies OR Outcome Study OR Studies, Outcome OR Study, Outcome OR Outcome Measures OR Measure, Outcome OR Measures, Outcome OR Outcome Measure.

### Study Selection and Data Extraction

Initially, the titles and abstracts of the studies were read according to eligibility criteria. Then, we fully read the studies that remained in order to extract data (titles, country, study design, population, sample size, method for assessing food consumption, UPF% total of energy intake, exposure variables, health outcomes, statistical analysis and adjustment methods, and main results). Two independent reviewers registered data in a previously structured Microsoft Excel spreadsheet. At this stage, we checked the reference list of studies included seeking for other potentially eligible studies.

### Data Synthesis Strategy

Data were extracted on study details using a structured form based on the research question. The following information was extracted from each study: author, year of publication, country, study design, study population/sample size, UPF exposure (dietary assessment methods, percentage of total energy consumed from UPF), health outcome (measures and data collection association to UPF exposure, and statistical analysis method) and main results. There was also no limitation on outcome categories. For a better presentation of the results, the articles included were divided according to life cycles: pregnant women, lactating women, and children (neonates, infants and children under 10 years old).

Outcomes were organized and tabulated by specific indicators: (a) Disease, defined as any disease, disorder or metabolic alterations or clinical condition cited; (b) Nutritional status, defined by overweight, obesity, adiposity, weight gain, body fat distribution (waist circumference), and relevant biomarkers for nutritional condition; (c) Feeding practices, included diet quality, unhealthy eating practices, duration of breastfeeding; and (d) Toxicity, used biomarker for it. Outcome measures comparison, effect sizes (HRs, ORs, and RRs) with 95% CIs were extracted.

### Assessment of Study Quality

The Newcastle–Ottawa scale was used to assess the methodological quality of retrospective studies applying the modified version (20) to assess cross-sectional studies (Risk of Bias Tool 2, RoB 2). The scale consists of eight questions; all are rated up to one star each, except for the question about comparability, which can be rated up to two stars. The version adapted for cross-sectional studies consists of seven questions rated up to one star each, except those on questions on comparability and outcome, which can be rated up to two stars. Total scores for both versions can reach a maximum of nine stars. A study with ≤6 points was considered of low quality and >6 points was considered of high quality.

The authors resolved mismatching opinions by consensus, and a third author was consulted to resolve conflicting interpretation, when necessary.

## Results

### Study Selection and Characteristics

**FIGURE 2:**
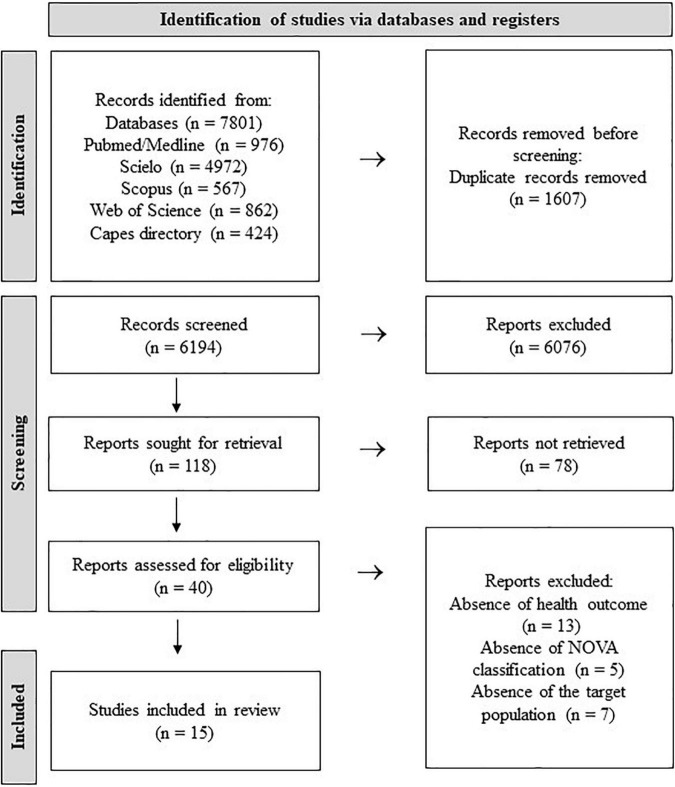
Flow diagram of database search and article eligibility.

The selection process identified 7,801 records in the databases, 6,194 were screened by titles after duplicates removed. Forty articles had their full-texts assessed for eligibility, being 15 of them (8 cohort and 7 cross-sectional studies) included into the systematic review (Figure 2). Of the 15 studies, nine were conducted in children ages <10 years, five in pregnant women, and one in lactating women. More than half of the studies included were carried out in Brazil (53.3%; *n* = 8) followed by European countries (*n* = 4) and two in the United States of America. Regarding the method of assessment of UPF intake, the food-frequency questionnaires (FFQs) was the most-used tool (*n* = 7), followed by 24-h Dietary Recall multiples (*n* = 6) (Table 2).

**TABLE 2:** Characteristics of included studies (*n* = 15).

Author(s), year, country	Study design	Population (number)	Assessment of UPF intake	UPF % total E intake- Relative exposure	Exposure variables	Health outcome(s) (study definition)	Statistical analysis method (adjustment)	Main results
Rohatgi et al. (13) United States	Cohort	Pregnant women (45)	152-item FFQ	54.4%	% of energy intake from UPF	Total GWG (Kg), neonatal percent body fat and site-specific skinfold measurements	Multiple linear regression - ANCOVA (age, race, weight status, socioeconomic status, physical activity, energy and fat intake)	A 1%-point increase in percent of energy intake from UPF was associated with a 1.33 kg increase in gestational weight gain (β = 1.3, 95%CI 0.3–0.4) and 0.22 mm increase in thigh skinfold (β = 0.2, 95%CI 0.005–0.4), 0.14 mm in subscapular skinfold (β = 0.1, 95%CI 0.02–0.3), and 0.62 percentage points of total body adiposity (β = 0.6, 95%CI 0.04–1.2) in the neonate
Silva et al. (14) Brazil	Cohort	Pregnant women (42)	FFQ (number of items not specified)	15.2%–16.9%	Total (Kcal) and % of energy intake from UPF	Total GWG (kg), HbA1c (%), 1-h postprandial glucose (mg/dL)	Multiple linear regression (age, for pre-gestational BMI, number of prenatal consultations, weekly weight gain in the third trimester, and number of visits with a nutritionist, parity and insulin doses)	The increase of every 1 kcal in the calorie intake from UPF in the third trimester increased glycated hemoglobin by 0.007% (β = 0.007, 95%CI 0.001–0.013), raised 1-h postprandial glucose by 0.14 mg/dL (β = 0.143, 95%CI 0.034–0.251), and added 0.01 kg to total gestational weight gain (β = 0.011, 95%CI 0.004–0.019)
Gomes et al. (22) Brazil	Cohort	Pregnant women (259)	>2 × 24 h-recall^1^	24.8%	% of energy intake from UPF	GWG per week (g)^6^	Multiple linear regression (skin color, pre-gestational BMI, work type, parity, socioeconomic status, education level)	A 1% point of increase in percentage of energy intake from UPF in the third gestational trimester led to an average increase of 4⋅17 g in weekly GWG (β = 4.17, 95% CI 0.55–7.79)
Borge et al. (31) Norway	Cohort	Pregnant women (77,768)	255-item FFQ	31.8%	UPF score	Child ADHD^3^ symptom score and ADHD diagnoses score	Bayesian linear regression (age, parity, pre-gestational BMI, education level, smoking and alcohol intake during pregnancy, maternal symptoms of depression and ADHD, child sex, child diet and child birth quarter)	A change of + 0.25 for the UPF score corresponding to relative change in mean ADHD symptom score of +3% (CI = 1.5, 4.5%). No reliable change in risk for the UPF score and ADHD diagnosis
Badanai et al. (23) Brazil	Cross-sectional	Pregnant women (784)	2 × 24 h-recall	33.4%	Tertiles of % energy intake from UPF	Feeling of depression or sadness (yes or no)	Adjusted logistic regression (age, education level, marital status, smoking, alcohol, physical activity, pre-gestational BMI, sleep, age gestational and total dietary energy)	Pregnant women ranked in the third tertile of % energy from UPF had a higher chance of feeling depressed or sad (OR = 2.39, 95%CI 1.29–4.41)
Amorim et al. (15) Brazil	Cross-sectional	Lactating women (294)	3 × 24 h-recall	16%	Tertiles of % energy intake from UPF	Breast milk vitamin E (BMVE) adequacy (>4 mgVE/780 mL), VE intakes (mg/day), serum alpha-tocopherol (μmol/L)	Multiple linear regression (age and socioeconomic status)	Lactating women ranked in the third tertile of % energy from UPF, the trajectories of BMVE adequacy reduced (β = –0.144, 95%CI-0.505, −0.063) and serum α-tocopherol reduced by 0.168 μmol/L (β = –0.168, 95%CI-0.047-0.010)
Costa et al. (24) Brazil	Cohort	Children 4–8 years (307)	2 × 24 h-recall	41.8%	% of energy intake from UPF (at age 4 years)	ΔBMI (Kg/m^2^), ΔWC (cm), Δwaist to height ratio (cm), Δskinfold sum, glucose (mmol/L), insulin (uU/mL) and HOMA-IR at age 8 years	Multiple linear regression (pre-gestational BMI, sex, birth weight, breastfeeding, family income, maternal education level and total screen)	UPF consumption at age 4 was associated with increased ΔWC from age 4 to 8 years (β = 0.07, 95%CI 0.01–0.13). No significant associations were observed for BMI, glucose profile and insulin resistance
Azeredo et al. (25)Brazil	Cohort	Children > 6 years (2,190)	2x < 88-item FFQ	42.3%	Quintiles of% energy intake from UPF	Wheeze, asthma, and severe asthma in past 12 months (yes or no)	Logistic regression (age, socioeconomic status, race, maternal variables, sex, parental smoking)	The highest quintile of UPF consumption at age 6, has no association with wheeze (OR = 0.85; 95% CI 0.54-1.34), asthma (OR = 0.84; 95% CI 0.58-1.21), or severe asthma (OR = 1.12; 95% CI 0.62-2.03) in early adolescence
Porto et al. (30) Brazil	Cohort	Children < 1 years (286)	11-item FFQ	–	UPF consumption ≥ 4 times per day	Exclusive breastfeeding duration (<120; 120–179; e ≥ 180 days)	Poisson regression (socioeconomic status, education level, marital status, parity, number of prenatal consultations, student)	Children who received breastfeeding exclusive for less than 180 days presented higher risk to early UPF introduction (PR = 2.17, 95%CI 1.09–4.30)
Chang et al. (26) England	Cohort	Children > 7 years (9,025)	3-day food diaries	23.2%Q1 –67.8%Q5	Quintiles of % energy intake from UPF	BMI-for-age (z score), BMI (Kg/m^2^), FMI (Kg/m^2^), total fat (%), LMI (Kg/m^2^), weight (Kg), fat mass (Kg), Lean mass (kg) and WC (cm)	Multiple linear regression (sex, age, race, birth weight, physical activity, maternal marital status, socioeconomic status)	Among those in the highest quintile of UPF consumption compared with their lowest quintile counterpart, trajectories of BMI increased by an additional 0.06 (β = 0.06, 95%CI 0.04–0.08) per year; FMI by an additional 0.03 (β = 0.03, 95%CI 0.01–0.05) per year; weight, by an additional 0.20 (β = 0.20, 95%CI 0.11–0.28) kg per year; and WC, by an additional 0.17 (β = 0.17, 95%CI 0.11–0.22) cm per year
Rauber et al. (21) United Kingdom	Cross-sectional	Children > 1.5 years (4,635)	3 or 4-day food diaries	76.0%	Quintiles of% energy intake from UPF	Excessive intake of free sugars (≥10% of total energy)	Poisson regression (age, sex, ethnicity, region, survey year and equivalized household income)	Children in the 5th quintiles of UPF consumption had a prevalence of excessive free sugar intake 60% higher (PR = 1.6, 95%CI 1.3–1.9) than those in the lowest quintile group.
Souza et al. (32) Brazil	Cross-sectional	Children 0–3 years (309)	35-item FFQ	–	UPF consumption ≥ 4 times per day	Non-cavited caries (white spots) and cavited caries (caries index)	Poisson regression (education level, duration breastfeeding status, caregiver age and use of dental services)	UPF consumption ≥ 4 times per day was associated with present both non-cavitated caries (PR = 2.25, 95%CI 1.19- 4.27) and cavitated caries (PR = 3.48, 95%CI 1.18- 10.30) compared with those who have consumed them up to 3 times a day.
Steelel et al. (27) United States	Cross-sectional	Children > 6 years (9,416)	1 × 24 h-recall	68.2% (6–11 years)	Quintiles of% energy intake from UPF	Sum of urinary biomarkers of phthalates and bisphenol compounds or their metabolites (ng/mL): Di(2-ethylhexyl) phthalate (ΣDEHP), Di-isononyl phthalate (ΣDiNP), Monocarboxynonyl phthal-ate (mCNP), Mono (3-carboxypropyl) phthalate (mCPP), Monobenzyl phthalate (mBzP), Bisphenol A (BPA), Bisphenol F (BPF) and Bisphenol S (BPS)	Multiple linear regression (ηmol/g creatinine, sex, age, race/ethnicity, family income, cycle, energy intake, BMI classification, physical activity)	The highest quintile of UPF intakes had 23.4% (95%CI 7.9–41.2) higher levels of ΣDiNP, 14.6% (95%CI 4.4–25.8) higher levels of mCNP, 11.5% (95%CI 0.2- 24.1) higher levels of mCPP, 10.7% (95%CI 0.6–23.3) higher levels of mBzP, 6.2% (95%CI −2.7–15.9) higher levels of BPA, and 33.8% (95%CI 11.7–60.3) higher levels of BPF.
Araya et al. (28) Chile	Cross-sectional	Children < 6 anos (960)	1 × 24 h-recall	49%	Quintiles of% energy intake from UPF	Nutrient composition of the diet (mean and standard deviation)	Multiple linear regression (sex, age, weight status, maternal education level, day of the dietary recall)	The 5th quintile of UPF intakes has higher energy, satured fats, monounsaturated fats, total sugar, vitamin D intakes and lower fiber, polyunsaturated fats, sodium, zinc, vitamin A and folate intakes than 1st quintile of UPF
Moreno-Galarragaa et al. (29) Spain	Cross-sectional	Children 4–5 years (513)	149-item FFQ	39.9%	Median of% energy intake from UPF (two groups: high and low)	Wheezing respiratory diseases - asthma and bronchitis (yes or no)	Logistic regression (age, sex, race, prenatal information, allergy, smoke exposition	A high consumption of UPF was associated with an increase of 87% in the prevalence of wheezing respiratory diseases (OR = 1.87; 95%CI 1.01–3.45). It was found that a higher consumption of UPF multiplied by 2.12 (95%CI 1.10–4.05) the prevalence of bronchitis/recurrent wheezing.

*FFQ, food frequency questionnaire; R, energy; ADHD, attention deficit hyperactivity disorder; BMI, body mass index; GWG, gestational weight gain; WC, waist circumference; FMI, fa mass index; LMI, lean mass index; BMVE, breast milk vitamin E; VE, vitamin E; Q, quintile; HOMA-IR, homeostatic model assessment of insulin resistance (insulin mU/ml x glucose mmol/l)/22.5).*

### Principal Findings

The contribution of UPF in the diet ranged from 15.2 to 76.0%, and higher levels of consumption were reported in children, especially English children (21). Exposure was assessed through UPF total energy contribution in twelve studies (13–15, 21–29) and selected studies examined the association between UPF consumption and the following health outcomes: weight gain (*n* = 4) (13, 14, 22, 26), adiposity measures gain (*n* = 3) (13, 24, 26), overweight/obesity (*n* = 2) (24, 26), nutrient intakes (*n* = 2) (21, 28), breastfeeding exclusive duration (*n* = 1) (30), alpha-tocopherol serum levels/nutrition composition of human milk (*n* = 1) (15), glucose levels (*n* = 1) (14), Attention deficit hyperactivity disorder (ADHD) (*n* = 1) (31), depression or sadness (*n* = 1) (23), wheeze/asthma (*n* = 1) (25, 29), caries (*n* = 1) (32) and urinary biomarkers of phthalates and bisphenol compounds levels (*n* = 1) (27). In the following topics we developed a narrative synthesis of our findings.

### Pregnant and Lactating Women

Three studies (13, 14, 22) found a positive association between UPF consumption and gestational weight gain (GWG) in the third gestational trimester, indicating that the 1% or Kcal point increased in the calorie intake from UPF increased total (Kg) or weekly (g) gestational weight gain.

Silva et al. (14) also found a significant positive association with glucose levels, indicating that high consumption of UPFs was associated with an increased glycated hemoglobin and postprandial blood glycemia in diabetic pregnant women. Badanai et al. (23) showed a higher chance of feeling depressed or sad (OR = 2.39, 95% CI 1.29–4.41) in the pregnant ranked in higher tertile of % energy from UPF consumption.

Only two cohort studies (13, 31) reported impact of UPF consumption during pregnancy on their neonates, even after adjustment for potential confounding factors. Rohatgi et al. (13) found that the UPF consumption was also related to increase of neonatal adiposity, with increase measurements of the thigh and subscapularis skinfold (mm). Rohatgi et al. described any limitations of study: the small sample size, the protocol and the database developed before the publication of the NOVA classification which means that the consumption of ultra-processed foods may have been underreported. One cross-sectional study with 77,768 Norwegian pregnant and their children (31) reported positive association between higher UPF score during pregnancy and increased ADHD symptoms score in children age 8 years using the Parent Rating Scale for Disruptive Behavior Disorders.

In lactating women, only one study was found regarding the researched parameters (15), and investigated the relationship between UPF maternal consumption and maternal vitamin E status. A higher dietary share of UPF (% energy) reduced serum concentration of alpha-tocopherol (vitamin E) (β = −0.168, 95% CI = −0.047–0.010) and the trajectories of probable inadequacy of the vitamin E content in breast milk (adjustment for income and maternal age), due to the lower availability of vitamin in the milk samples analyzed, suggesting that a higher dietary share of UPF may cause inadequate vitamin E status in lactating women and, possibly, low values in breast milk.

### Children

Nine of the 15 studies included in this review were carried out with children (21, 24–30, 32) in different age groups, only three (32.2%) had children under 36 months of age as their population; and four reported the UPF consumption in children over 6 years. The selected studies were conducted in Brazil (*n* = 4), Chile (*n* = 1), England (*n* = 1), Spain (*n* = 1), in the United Kingdom (*n* = 1), and in the United States of American (*n* = 1).

The contribution of UPF in the diet ranged from 41.8 to 76.0% of the total calories, being higher in children over 1.5 years in the United Kingdom (21). One cohort study (30) analyzed the impact of the introduction of four or more UPF in the first year of life and exclusive breastfeeding (EBF) and found that children who received EBF for less than 180 days presented higher risk to early UPF introduction (PR = 2.17, 95%CI 1.09–4.30).

Two cohort studies (24, 26) found a positive association between adiposity measurements (body mass index-BMI, weight, fat mass index, waist circumference) and high UPF consumption. A prospective birth cohort study (Avon Longitudinal Study of Parents and Children-ALSPAC) in England followed up children from 7 to 24 years of age during 20 years and investigated the impact UPF consumption in the adiposity trajectories from childhood to early adulthood. In a sample of 9,025 children over 7 years followed up for 10 years, every adiposity indicator increased per year, showing a greater and faster progression of these in the group of individuals in the highest UPF consumption quintile when compared to the group in the 1st quintile (26). Costa et al. (24) reported that UPF consumption at age 4 years was a predictor of an increase in delta waist circumference from age 4 to 8 (β = 0.07; 95%CI 0.01–0.14) but no significant associations were observed for BMI and glucose metabolism.

Two of the studies included (21, 28) found an association between UPF consumption and diet nutritional quality. A higher UPF consumption resulted in more energy, fats, carbohydrates, total sugars, and vitamin D intakes, and indicated a negative association with the consumption of proteins, polyunsaturated fats, fiber, zinc, vitamin A, and sodium (28). Rauber et al. (21) reported that UPF account 64.7% of total free sugars in the United Kingdom diet and 74.94% of children aged >1.5 years had excessive free sugar intake, indicating increase linearly across quintiles of UPF consumption (PR = 1.6, 95%CI 1.3–1.9).

We identified three studies on children investigating UPF exposure and disease. The Brazilian cohort study (25) found no association between UPF consumption in childhood (age 6 years) and wheeze, asthma or severe asthma in adolescence. In contrast, in Spanish children aged 4–5 years, a high UPF consumption was associated with an increase of 87% in the prevalence of wheezing respiratory diseases (OR = 1.87, 95%CI 1.01–3.45) and increased in two times the prevalence of bronchitis/recurrent wheezing. Other study showed an increased UPF consumption greater than or equal to 4 times a day was associated with cavitated caries (deteriorated surfaces) and non-cavitated caries (active and inactive white spots) in early childhood (32), indicating that beyond the presence of saccharose, other foods that contain fermentable carbohydrates, such as some UPF, are retained in the teeth for longer, facilitating the development of caries (33).

Steelel et al. (27) identified that a greater participation of UPF in the diet was associated with urinary concentration of phthalates and bisphenol F (BPF) biomarkers. Phthalate and bisphenol are chemicals present in materials such as plastic, paper, metal and glass, used in food packaging. There was no association of UPF consumption with bisphenol A (BPA) and an inverse association with bisphenol S (BPS) was observed.

### Methodological Quality

The methodological quality assessment of the fifteen studies included in this systematic review is presented in Table 3. The scores were distributed into three domains as previously shown in the scale: selection, comparability and outcome. The average by the Newcastle-Otawa Scale was 7 (6–9) stars. The most common bias risk was incomplete representativeness (13, 14, 22, 23, 26, 27, 29). Out of the studies included, 14 presented high methodological quality, while only one (23) had a score of less than 6 stars.

**TABLE 3:** Methodological quality assessment and characteristics of the studies included in the review.

Cohort – Newcastle-Ottawa Scale (*n* = 8)										

	**Selection**				**Comparability**	**Outcome**				**Total score**
**Quality criteria**	**1. Representativeness of the exposed cohort**	**2. Selection of the non-exposed cohort**	**3. Ascertainment of exposure**	**4. Demonstration that outcome of interest was not present at start of study**	**1. Comparability of cohorts on the basis of the design or analysis**	**1. Assessment of outcome**	**2. Was follow-up long enough for outcomes to occur?**		**3. Adequacy of follow-up of cohorts**	
Rohatgi et al. (13)			*	*	**	*	*		*	7
Silva et al. (14)			*	*	**	*	*		*	7
Gomes et al. (22)		*	*	*	**	*	*			7
Borge et al. (31)	*	*	*	*	**	*	*			8
Costa et al. (24)	*	*	*	*	**	*	*			8
Azeredo (25)	*		*	*	**	*	*			7
Porto et al. (30)		*	*	*	**	*	*		*	8
Chang et al. (26)		*	*	*	**	*	*			7

**Cross-sectional Newcastle-Ottawa Scale Modified (*n* = 7)**										

	**Selection**				**Comparability**	**Outcome**				**Total score**
	**1. Representativeness of the sample**	**2. Sample size**	**3. Ascertainment of exposure**	**4. Non-responders**	**1. The subjects in different groups of results are compatible based on the study design and in the analysis. The base factors are controlled.**	**1. Evaluation of the results**		**2. Statistical test**		

Amorim (15)	*	*	*	*	**	*		*		8
Badanai et al. (23)			*	*	**	*		*		6
Rauber et al. (21)	*	*	*	*	**	*		*		8
Steelel et al. (27)	*	*	*	*	**	*		*		8
Souza et al. (32)		*	*	*	**	*		*		7
Araya et al. (28)	*	*	*	*	**	*		*		8
Moreno-Galarragaa et al. (29)		*	*	*	**	*		*		7

*Pontuation.

## Discussion

Although the relevance of increased UPF consumption is associated with a higher incidence of adverse health outcomes in adults and elderly, especially in Western countries, the findings of this review indicate few studies correlating UPF consumption with health outcomes in the maternal-infant group, with an analysis in childhood being predominant. This may be a reflection of the influence of the food industry on infant feeding, with the high availability in the market of industrialized infant foods predominantly of the UPF type (34–36).

Its greater participation with advancing age, especially in school age, associated with lower maternal education and lower income (37, 38), once that the family food environment is also determinant for UPF consumption in childhood (16). All these determining factors become an important warning to reinforce the need for adequate mother and infant nutrition and to understand its repercussions beyond childhood, into adulthood.

This greatest participation of UPF was observed in studies that analyzed infants diets (21, 24–30, 32), representing up to 70% of calories consumed by children older than 1.5 years (21) which is much higher than the average found in pregnant women (22.6%) and lactating women (16%).

Currently, UPF represent more than 60% of the daily caloric intake among school-age children in the United Kingdom and in the United States (39, 40). A populational study with Brazilian children found that 74% of children under 24 months had already consumed some type of UPF (41). UPF early exposure may cause a low diet quality, since these food products have lower amounts of fiber, vitamins and higher energy density, free sugars, total fats and sodium (29).

**FIGURE 3:**
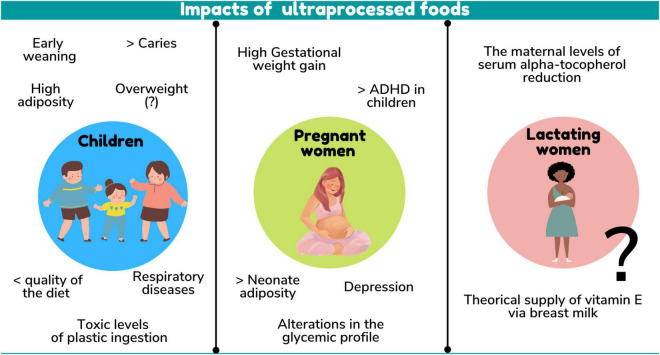
Impacts of ultraprocessed foods.

There were several health outcomes in children related to UPF consumption. A relationship was found both with the nutritional quality of the diet and with excess weight, adiposity, changes in the lipidic profile, inadequate dietary practices, respiratory diseases, dental caries and even toxicity related to ingestion of plastics present in UPF packaging (Figure 3).

Excess weight and adiposity are associated with an increased risk of related comorbidities with the development of non-communicable diseases in adulthood, which may result in decreased life expectancy, loss of productivity and increased costs to the health system (5). Some studies did not find a relationship between UPF consumption and overweight, probably due to their cross-sectional design or to a high rate of UPF consumption in more than 50% of the participants, regardless of BMI, suggesting that cohort studies are needed to better understand UPF consumption and the development of obesity (28).

Crimarco and collaborators, trying to explain how UPF consumption influences excess weight and adiposity, concluded that UPF consumption led to increased energy intake and weight gain compared to whole foods, that is, UPF high energy density is the main determining factor for this association. This fact can be extended to the analysis of this association in childhood, since UPF targeted at infants also have a high density compared to unprocessed/minimally processed foods (34, 36) and may cause low dietary quality (28).

However, one study did not find any effect on the outcomes studied (28), which may be attributed to the high consumption of UPF in the evaluated children, regardless of the BMI classification, high age range (2–10 years), and insufficient amount of food survey applied to identify consumption patterns.

Furthermore, it is also suggested that null results may be attributable to factors associated with the etiology of obesity, such as physical activity, genetics and family lifestyle, which were not evaluated in the studies. This divergence highlights the need to carry out clinical trials to assess whether reducing UPF consumption among children can reduce the prevalence of overweight and obesity.

Interestingly, only one study assessed the impact on breastfeeding children’s health, finding that the introduction of 4 or more UPF had a negative impact on EBF duration, increasing the chances of negative health outcomes for these children (24).

Breastfeeding provides benefits for both mother and child. Children who are breastfed for longer periods have a lower risk of being affected by infectious diseases, mortality, dental problems and have better levels of intelligence quotient (IQ) than those who are breastfed for shorter periods, or not breastfed (41, 42). Increasing evidence also suggests that breastfeeding may protect against overweight and diabetes. In lactating women, breastfeeding may prevent breast cancer, increase interval between births and reduce the risk of a woman developing diabetes or ovarian cancer (43).

Respiratory diseases are increasingly frequent in childhood – asthma being the most common chronic disease in developed countries. Despite its genetic basis, exogenous factors such as diet and breastfeeding can influence and knowledge of these modifiable factors may help in the development of primary prevention strategies and contribute to achieving better control of the symptoms of this disease (44, 45). From this perspective, the results on the association of UPF consumption and respiratory diseases were divergent. In school-age children, the prevalence of wheezing diseases reached 87% of the cases, with the highest participation of these foods in the diet (29). Another study found no association between UPF consumption at 6 years of age with the presence of asthma and wheezing among adolescents, demonstrating the need for studies in other populations, mainly due to some limitations in the consumption analysis, as reported by the authors themselves (30).

Another disease found to be associated with UPF consumption was the development of early childhood caries (27). Dental caries is the most prevalent chronic disease in preschool children worldwide and has serious negative impacts on quality of life, as well as high treatment costs for the family and society (46). This association is probably due to the high free sugars content in these foods, as can be seen in a study involving 3,427 industrialized foods targeted at children found on the market in 27 European countries (35). Interventions with the objective of reducing UPF consumption must be implemented to improve children’s oral health, as they present a higher UPF consumption percentage.

In addition to the harmful effects on health caused by the UPF consumption, it is worth mentioning that these food products are often packaged in materials considered sources of endocrine disruptors, such as phthalates and bisphenol, associated with adverse health outcomes, especially in periods of high cell development (47).

Cross-sectional studies specifically link exposure to Bisphenol A (BPA) with increased risk of diabetes, general and abdominal obesity and hypertension, whereas phthalates are shown to be related to diabetes and insulin resistance (48). The intake of aluminum can be associated with neurodegenerative diseases due to its higher concentration in food additives, flavorings and packaging materials, with possible health consequences in adults and children (49–51).

Elevated levels of toxic compounds from plastics in urine were observed in children monotonically from the lowest to the highest quintile of UPF consumption, which may pose a long-term health risk of exposure given the high percentage of UPF targeted at children (32) and the early exposure of children to this type of product (40).

The health of the mother-infant dyad and, consequently, the child’s growth and development may be positively impacted by a healthy maternal diet in the pre-gestational, gestational and lactation phases (52–55). On the other hand, in pregnancy and lactation, maternal intake of fat and added sugar may be associated, respectively, with an increase in infant adiposity and greater infant weight/length at 6 months of life (56), demonstrating the adverse impact of maternal UPF consumption on postnatal growth. Therefore, maternal UPF consumption may result in greater total body adiposity in infants and, according to the study by Borge et al. (31), which followed children up to 8 years of age, may even result in an increasing trend of ADHD symptoms in children.

Although the possible impact of maternal dietary quality during pregnancy on child neurodevelopmental functions is still poorly investigated, current evidence reports that the intrauterine environment may be a trigger for the development of neurodevelopmental disorders and that foods high in fat, free sugars and chemical additives are being linked to neuroinflammation and reduced cognitive function. These unfavorable outcomes for child health become an important warning for the reduction of UPF consumption during pregnancy (57–60). Despite little evidence regarding childhood, UPF consumption during pregnancy was associated with unfavorable health outcomes in pregnant women, such as high weight gain in the third trimester of pregnancy, increased glycemia and depression (14, 22, 31).

All these findings can be related to the UPF low nutritional quality. High weight gain due to the high energy density of these foods can lead to the risk of complications in pregnancy such as gestational hypertensive diseases, gestational diabetes, premature birth, need for cesarean section and macrosomia (61, 62) Glycemic control is also impaired due to the excess and low quality of carbohydrates found in processed foods (13). The findings of gestational depression indicate that psychosocial adaptation is another aspect that may be linked to the pregnant woman’s diet. It is recognized that commensal bacteria in the intestine may have direct and indirect effects on cognition and behavior (62). Studies have identified possible relationships between a UPF-rich diet, dysbiosis and the development of neuroinflammation, which may affect pregnancy through its effect on maternal mental health (60, 63).

Of the three life cycles studied, the lactation phase is practically invisible to the state of the art between UPF consumption and maternal and child health, with only one study being found addressing UPF consumption by lactating women and the micronutrient status and composition of breast milk. In this study, it was observed that a greater participation of UPF in the diet can negatively impact maternal serum alpha-tocopherol levels and the theoretical supply of vitamin E to the infant via breast milk analyzed (15), which alerts for the development of studies to assess the impact of UPF consumption on the composition of breast milk and on the development of specific nutritional deficiencies during lactation. This downward trend in micronutrients was observed in Brazil’s 2008–2009 Household Budget Survey, which found that increased dietary UPF was associated with reduced content of vitamins B12, D, E, niacin and pyridoxine, and copper, iron, phosphorus, magnesium, selenium, and zinc, when compared with fresh and minimally processed foods (64).

Overall, this was the first systematic review that gathered information on the impact of UPF consumption on maternal and child health, demonstrating that there is still a need for further studies to clarify the effect of these foods in different populational groups, especially in lactation, that use appropriate dietary assessment tools to analyze UPF consumption and that investigate the different health and nutrition outcomes in childhood, pregnancy, and lactation. The strengths of studies were in nationally representative samples (*n* = 6) or large samples (*n* = 7) with data extraction at individual intake, except to Porto et al. (30) and Souza et al. (32), and outcome measurements by trained personnel in all studies. The studies used statistical analysis methods with adjustment and demonstrated group comparison of higher to lower UPF exposure (*n* = 8), followed by results according to % of energy intake from UPF (*n* = 4), UPF consumption per day (*n* = 2) and UPF score (*n* = 1). In several instances, foods were classified differently across surveys due to different contexts. For example, some FFQs had few or did not specify food items and were not developed for UPF analysis (14, 30, 32). Additional complexities included application to food databases with a lack of information on the differentiation of canned food into processed food (PF) or UPF, databases disaggregating foods to nutrient content rather than processing type (e.g., cake disaggregated to component ingredients could inadvertently be classed as PF, and not correctly as UPF if it were ultra-processed), and disaggregating handmade dishes (e.g., pizza) into major food-items in the recipe (e.g., group 1) rather than underlying ingredients (flour, cheese, meat, sauce, salt, and oil), which is the recommended approach.

It is noteworthy that, despite studies being scarce, out of the 15 studies included in the review, only three did not find an association between UPF consumption and health outcomes, demonstrating that these foods also have an important public health impact in the maternal and child group. Among the outcomes found, the association between UPF and the increase of weight, adiposity measures and diseases or metabolic changes and profile diet in childhood and pregnancy related to non-communicable diseases stand out. Factors associated with the prevention of overweight, such as support for increasing the duration of breastfeeding and decreasing maternal UPF consumption, are also important for planning health policies to reduce childhood obesity (65). This dataset can help in future nutritional recommendations for this population.

Since NOVA was established, UPF have become dominant components in the diets of populations across the world (4), contributing with over than 50% of energy intake in high-income countries, such as Australia, the United Kingdom, and the United States (18, 21, 66, 67) and up to 30% in low- and middle-income countries, such as Brazil, Chile, and Mexico (10, 68, 69, 71), with consumption volumes increasing rapidly (70). As middle-income countries are home to the vast majority of the world’s population, understanding the implications of increased UPF consumption for global human health is of utmost importance.

This study has some limitations. The current analysis is limited to the data currently available. The limited number of studies with infants, wide variation between existing instruments to assess UPF intake – often quite limited in scope and assessing only one aspect (i.e., cumulative UPF consumption). Finally, we suggest that the weaknesses observed in the studies included in this systematic review are addressed in future studies evaluating UPF consumption and its impacts on maternal-child health.

## Conclusion

Despite little evidence in the phase of lactation and pregnancy, the selected studies showed a negative impact on health associated with the high UPF consumption in the three cycles of life, which reflected in different aspects. The repercussions are seen in several spheres, more importantly on nutritional indicators (overweight, adiposity measures, low levels of serum alpha-tocopherol and in breast milk, inadequate dietary practices, lower nutritional quality of the diet), but also on metabolic alterations (high glycemic levels and lipid profile), presence of diseases (depression, ADHD, caries, and respiratory diseases), and toxicity (high levels of toxic compounds from plastics in the urine).

Given the increased participation of UPF in the diet, the importance of carrying out more studies to investigate their effects on the health of different populations is highlighted, especially during the lactation phase, whether in infants or lactating women, and their impact on the composition of the breast milk, in view of the large gap on this regard. Thus, information based on scientific evidence is important for the promotion of policies and programs to encourage health promotion that encourage healthy and adequate eating practices, which provide the necessary conditions for access to food fitting for the maintaining, improving or restoring health, mainly in life cycles of great biological vulnerability, such as maternal and child health.

## Data Availability Statement

The raw data supporting the conclusions of this article will be made available by the authors, without undue reservation.

## Author Contributions

PO was the main author, reviewer, participated in the data extraction, preparation, and writing of this article. JS was the second reviewer, participated in the data extraction, preparation, and writing of the article. DA participated in the writing of this article, especially in detailing the methodology, and analyzing the quality of the studies. EA participated in the writing of this article, especially in detailing the results, and conclusion. KR was the third reviewer, creator, and advisor of this work, participated in the analysis of results, and writing and final review of this article. JD and DB participated in the writing and final review. All authors contributed to this article and approved the submitted version.

## Conflict of Interest

The authors declare that the research was conducted in the absence of any commercial or financial relationships that could be construed as a potential conflict of interest.

## Publisher’s Note

All claims expressed in this article are solely those of the authors and do not necessarily represent those of their affiliated organizations, or those of the publisher, the editors and the reviewers. Any product that may be evaluated in this article, or claim that may be made by its manufacturer, is not guaranteed or endorsed by the publisher.
